# Des fractures spontanées récidivantes

**DOI:** 10.11604/pamj.2014.18.73.3147

**Published:** 2014-05-23

**Authors:** Faida Ajili, Ikdam Blouza

**Affiliations:** 1 Service de Médecine Interne, Hôpital Militaire de Tunis, Montfleury 1008, Tunis, Tunisie; 2 Service de Stomatologie, Hôpital militaire de Tunis, Montfleury 1008, Tunis, Tunisie

**Keywords:** Fractures spontanées, densitométrie osseuse, ostéocondensation, ostéopétrose, spontaneous fractures, bone density, osteocondensation, osteopetrosis

## Image en medicine

Patiente âgée de 21 ans adressée pour des abcès dentaires récidivants et victime depuis l'âge de six ans de multiples fractures des os des 4 membres. Elle a 2 frères ayant fait chacun des fractures osseuses multiples. Elle avait un retard staturo-pondéral et des déformations des os des membres inférieurs (A). Son morphotype se caractérisait par un aspect triangulaire au visage. L'examen de la bouche retrouvait une issue de pus de la molaire inférieure gauche. Les bilans inflammatoire et phospho-calcique étaient normaux. Les radiographies des membres inférieurs avaient montré une déformation osseuse (B), des cals osseux et du matériel d'ostéosynthèse. Par ailleurs, on a retrouvé une ostéocondensation diffuse avec un aspect en “loup de carnaval” du massif facial (C) et de “vertèbres en sandwich” sur la radiographie du rachis lombaire de profil (D). La densitométrie osseuse avait montré un T-score élevé à + 3,9 DS au niveau lombaire. Le scanner du massif facial a conclut à une ostéomyélite mandibulaire chronique. Devant ce tableau clinico-radiologique, on a éliminé les maladies ostédystrophiques: l'ostéogenèse imparfaite, l'ostéoporose juvénile idiopathique, le syndrome Ostéoporose généralisée et pseudogliome et l'hypophosphatasie. Ainsi, le diagnostic d' « Ostéopétrose » dans sa forme autosomique dominante compliquée par une ostéomyélite mandibulaire a été retenu devant la densité osseuse augmentée et les descriptions radiologiques. Une double antibiothérapie a été instituée associée à un débridement de la nécrose osseuse et à des séances d'oxygénothérapie hyperbare. L'évolution est faite par une amélioration clinique avec un recul de 12 mois.

**Figure 1:**
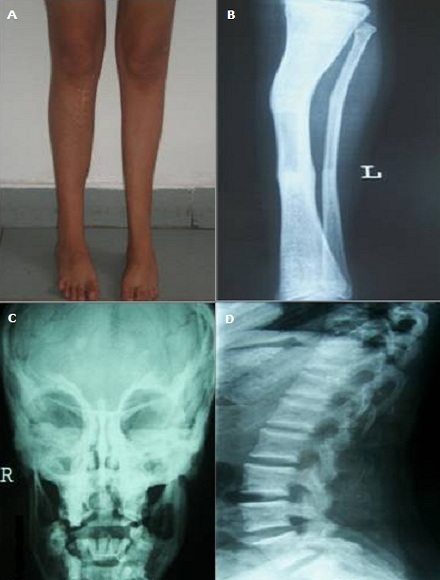
A) Déformations des os des membres inférieurs à type de genu valgum et pieds en varus équin à gauche et valgus du pied droit; B) Radiographies des membres inférieurs montrant des cals osseux avec du matériel d'ostéosynthèse; C) Radiographie de la face montrant un aspect en “loup de carnaval” du massif facial; D) Aspect de “vertèbres en sandwich” sur la radiographie du rachis lombaire de profil

